# Human surface ectoderm and amniotic ectoderm are sequentially specified according to cellular density

**DOI:** 10.1126/sciadv.adh7748

**Published:** 2024-03-01

**Authors:** Shota Nakanoh, Kendig Sham, Sabitri Ghimire, Irina Mohorianu, Teresa Rayon, Ludovic Vallier

**Affiliations:** ^1^ Wellcome-MRC Cambridge Stem Cell Institute, Jeffrey Cheah Biomedical Centre, University of Cambridge, Cambridge CB2 0AW, UK.; ^2^ Epigenetics & Signalling Programmes, Babraham Institute, Cambridge CB22 3AT, UK.; ^3^ Berlin Institute of Health Centre for Regenerative Therapies, Charité - Universitätsmedizin Berlin, Berlin 13353, Germany.; ^4^ Max Planck Institute for Molecular Genetics, Berlin 14195, Germany.

## Abstract

Mechanisms specifying amniotic ectoderm and surface ectoderm are unresolved in humans due to their close similarities in expression patterns and signal requirements. This lack of knowledge hinders the development of protocols to accurately model human embryogenesis. Here, we developed a human pluripotent stem cell model to investigate the divergence between amniotic and surface ectoderms. In the established culture system, cells differentiated into functional amnioblast-like cells. Single-cell RNA sequencing analyses of amnioblast differentiation revealed an intermediate cell state with enhanced surface ectoderm gene expression. Furthermore, when the differentiation started at the confluent condition, cells retained the expression profile of surface ectoderm. Collectively, we propose that human amniotic ectoderm and surface ectoderm are specified along a common nonneural ectoderm trajectory based on cell density. Our culture system also generated extraembryonic mesoderm–like cells from the primed pluripotent state. Together, this study provides an integrative understanding of the human nonneural ectoderm development and a model for embryonic and extraembryonic human development around gastrulation.

## Teaser

The in vitro model reveals that human nonneural ectoderm develops into embryonic and extraembryonic tissues based on cellular density.

## INTRODUCTION

A comprehensive understanding of human development at the cellular and molecular levels is crucial for advancing both basic research and therapeutic applications. Owing to the limited access to human embryos, developmental biology has relied on animal models to uncover the general principles of embryogenesis (*1*). Nonetheless, interspecies comparisons have revealed species-specific traits in developmental processes, highlighting the necessity to directly study human development (*2*). Extraembryonic tissues show marked variations between humans and other mammalian species, such as mice. This is exemplified by differing competencies for trophectoderm fate, modes of amniogenesis, and origins of extraembryonic mesoderm (*3*–*7*). Alternatively, in vitro differentiation of human pluripotent stem cells (hPSCs) derived from embryos or by cellular reprogramming (*8*, *9*) has provided important insights into the mechanisms controlling lineage commitment in human embryos (*10*–*13*). Furthermore, recent progress in stem cell–based embryo models has revealed that hPSCs retain self-organizing capacities to recapitulate human embryogenesis (*14*–*18*).

Amnion is an extraembryonic membrane enclosing the embryo/fetus in amniotic fluid, which fostered the key evolutionary innovation for amniotes to adapt to the terrestrial environment (*19*). In humans and macaque monkeys, cells emerging from pluripotent epiblast before and early in gastrulation (amnioblasts) form the monolayered amniotic ectoderm (AE), which is later outlined by the amniotic mesoderm to establish the bilaminar definitive amnion (*20*, *21*). AE develops through three stages (*20*): (i) The intercellular space within the epiblast mass expands eccentrically. (ii) The epiblast cell layer adjacent to the cytotrophoblast becomes thin and transiently opens to form a tropho-epiblastic cavity. (iii) The cells at the edge of the epiblastic disc upfold and spread to enclose the cavity. It has been reported that hPSCs give rise to cells similar to amnioblasts (*22*, *23*), whereas naïve hPSCs generate distinct amniotic cells which seem to contribute to the early stages of AE formation (*24*). Hallmark genes for human AE, such as *ISL1*, *GABRP*, *VTCN1*, and *WNT6*, have been identified by descriptive studies of early mouse definitive amnion, monkey AE, human amnion at 9 to10 weeks of gestation, and hPSC-derived amniotic sac models (*22*, *25*–*27*). Among these genes, a LIM-domain transcription factor, *ISL1*, was shown as an early marker of primate amnioblasts critical for their maturation (*28*).

On the other hand, surface ectoderm (SE) is a continuous dense sheet of epidermal progenitors, which begin to appear around gastrulation and eventually give rise to the skin and epithelia of the eye, mouth and nasal cavity, as well as their appendages (*29*). SE originates from a subpopulation of ectoderm that is set apart from neural commitment by the action of bone morphogenetic protein (BMP) signaling, whereas ectoderm derives from pluripotent epiblast that is neither exposed to fibroblast growth factor (FGF) nor NODAL signaling, both of which promote commitment to mesoderm and endoderm (*30*, *31*). Recently, single-cell RNA sequencing (scRNA-seq) analyses revealed that AE and SE largely share the expression of nonneural ectoderm (NNE) genes (*24*, *32*). Protocols to differentiate hPSCs toward AE and SE use similar signaling cues—activation of BMP signaling and attenuation of mesendoderm specification achieved by FGF/extracellular signal–regulated kinase (ERK) inhibition, NODAL/SMAD inhibition, and/or gamma-secretase inhibition (*4*, *5*, *33*–*35*). Therefore, the molecular signatures of human AE and SE are still elusive, and the biological significance of their resemblance as well as the mechanisms to separate these lineages are not yet elucidated.

To address these questions, we developed an hPSC culture system named ABCP after Activin A, BMP4, GSK3 inhibitor (CHIR99021: CHIR), and FGF/ERK signal inhibitor (PD0325901: PD). ABCP culture effectively induced the expression of AE markers. Single-cell transcriptome analyses of the in vitro differentiation detected a population presenting SE profile (up-regulation of NNE genes but not AE genes) and indicated AE specification through an SE-like state. In addition, cells grown in ABCP culture selectively up-regulated NNE genes when the seeding density was high. These results suggest a sequential differentiation of SE and AE restrained by high cellular density. We also described the emergence of human extraembryonic mesoderm in ABCP culture, which has so far only been derived from naïve hPSCs (*6*).

## RESULTS

### MEK/ERK inhibition during mesendoderm specification activates amnioblast genes

**Fig. 1.**
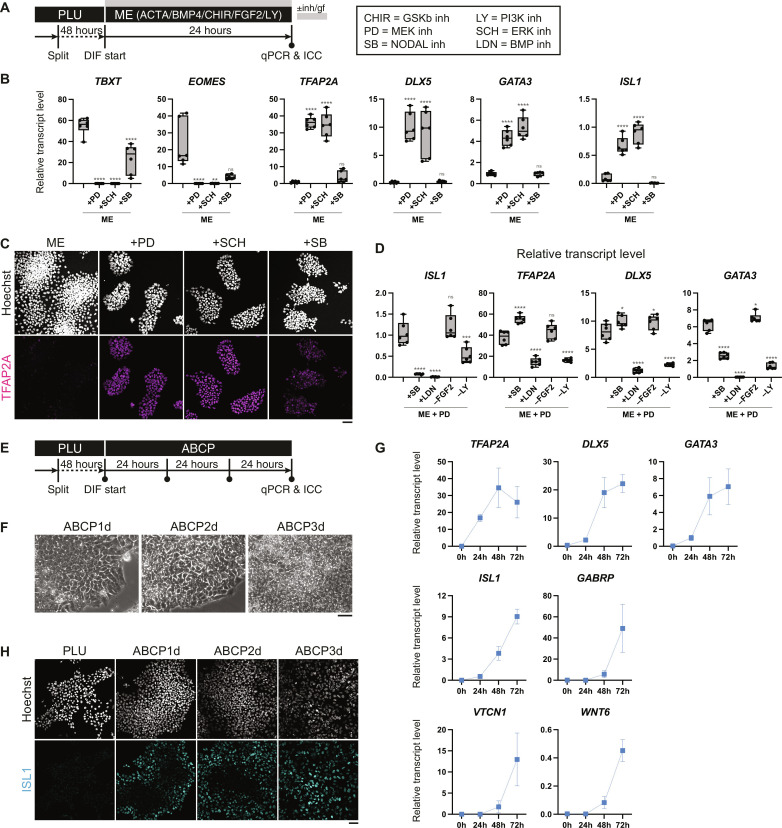
Establishing culture condition to induce amnioblast-like cells from hPSCs. (**A**) Diagram showing the culture schedule for (**B** to **D**) and description of the used inhibitors. PLU, pluripotency maintenance condition. (B) Quantitative gene expression analyses of H9 cells cultured for 24 hours in mesendoderm-induction conditions. Box-plot elements: The center line is the median; box limits are upper and lower quartiles; whiskers are minimum and maximum. Ordinary one-way analysis of variance (ANOVA) and the Kruskal-Wallis test were performed on the basis of the results of the Kolmogorov-Smirnov test. ns: *P* > 0.05, *P < 0.05, **P < 0.01, ***P < 0.001, and ****P < 0.0001. *n* = 6. (C) Immunocytochemistry of H9 cells cultured for 24 hours in different mesendoderm-induction conditions. Scale bar, 50 μm. (D) Quantitative gene expression analyses to optimize the culture condition by removing elements of the mesendoderm-induction condition with PD. Box elements and statistical analysis are as described in (B). *n* = 6. (**E**) Diagram showing the culture schedule over 3 days of ABCP culture for (**F** to **H**). (F) Bright-field micrographs. Scale bar, 50 μm. (G) Dynamic quantitative gene expression analyses over ABCP culture. Dots and error bars represent means and SDs, respectively. *n* = 3. (H) Confocal microscopy of ISL1 immunostaining. Scale bar, 50 μm.

We previously observed the up-regulation of NNE genes under inhibition of MEK by PD during mesendoderm differentiation of hPSCs (ME) promoted by Activin A, BMP4, CHIR, FGF2, and PI3K inhibitor (LY294002: LY) (Fig. 1A) (*32*). Similarly, an ERK inhibitor, SCH772984 (SCH), induced NNE genes, such as *TFAP2A*, *DLX5*, and *GATA3* (*36*–*38*) while reducing mesendoderm markers, *TBXT* (also known as *BRACHYURY*) and *EOMES* (Fig. 1B) (*39*). Thus, MEK/ERK signaling could have a decisive role in cell fate choice between mesendoderm and NNE. These NNE markers were not induced by a NODAL/SMAD signaling inhibitor, SB431542 (SB) (Fig. 1B). These results were confirmed at the protein level by immunofluorescence staining (Fig. 1C). We also found that the MEK/ERK inhibitors, but not the NODAL inhibitor, induced the expression of the amnioblast marker, *ISL1*, within 24 hours of treatment (Fig. 1B). To further identify critical signaling pathways in AE differentiation, we added specific inhibitors to or omitted the supplements from the ME+PD condition (Fig. 1D). *ISL1* was strongly suppressed by the additional inhibitions of the NODAL and BMP signaling by SB and LDN193189 (LDN), respectively, suggesting pivotal roles of these pathways in amnioblast specification (Fig. 1D). In contrast, the removal of FGF2 and LY enhanced *ISL1* expression level and cell survival, respectively (Fig. 1D). We therefore decided to dismiss these components. Removal of CHIR caused cell death and thus CHIR was kept. The resulting culture system was named ABCP after its supplements (Activin A, BMP4, CHIR, and PD). Following these promising results, we further examined the amnioblast-like cells generated in ABCP condition for an extended period (Fig. 1E). Cells kept in ABCP culture spread as squamous epithelial sheets (Fig. 1F). Quantitative reverse transcription polymerase chain reaction (RT-PCR) analysis detected the rapid and robust up-regulation of NNE markers in 48 hours of ABCP treatment and the marked increase of all the examined amnioblast markers toward 72 hours (Fig. 1G). Immunofluorescence staining revealed that ISL1 protein expression starts from the edges of colonies within 24 hours and progresses toward the center of the colonies (Fig. 1H). These results demonstrate that the ABCP culture system efficiently induces NNE and AE gene expression from hPSCs.

### Amnioblast-like cells in ABCP culture serve as a signaling center to provide gastrulation-inducing cues

**Fig. 2.**
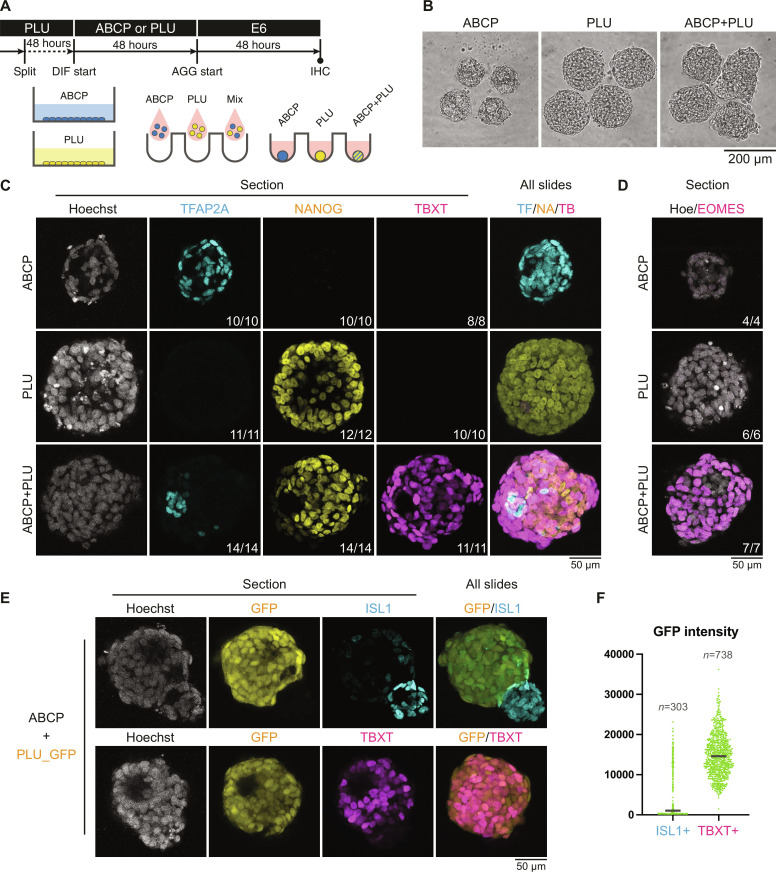
Functional analysis of hPSC-derived amnioblast-like cells in three-dimensional aggregations. (**A**) Diagram showing the culture schedule of ABCP induction and following aggregation formation. (**B**) Bright-field micrographs of aggregates formed using cells from the indicated conditions. (**C** and **D**) Confocal microscopy of whole-mount immunohistochemistry of aggregates. Fractions indicate the counts of aggregates with the represented staining patterns among all observations. (**E**) Confocal microscopy on whole-mount immunohistochemistry of aggregates using green fluorescent protein (GFP)–hPSCs. (**F**) GFP intensity of ISL1- and TBXT-positive cells in ABCP+PLU_GFP aggregates. Bars indicate medians.

Previous reports suggest that the primate AE acts as a signaling center to trigger primitive streak formation in the pluripotent epiblast (*23*, *28*). Thus, we tested if the amnioblast-like cells generated in ABCP culture could differentiate hPSCs into mesendoderm. hPSCs were grown in ABCP condition for 48 hours and then aggregated on low-adherent U-bottom 96-well plates (Fig. 2A). After an additional 48 hours in an E6 basal medium, cells from ABCP condition formed aggregates with multiple lumina, whereas cells from the pluripotency maintenance condition (PLU) solely formed packed spheres (ABCP and PLU, respectively; Fig. 2B). When the ABCP and PLU cells were mixed up together, the aggregates exhibited uneven shapes (ABCP+PLU; Fig. 2B). Immunofluorescence staining demonstrated that ABCP aggregates expressed TFAP2A but not NANOG, while PLU aggregates were negative for TFAP2A and positive for NANOG (Fig. 2C, top and middle rows). In line with these observations, ABCP+PLU aggregates were partially stained for both markers in a mutually exclusive manner (Fig. 2C, bottom row). In contrast, the primitive streak marker, TBXT/BRACHYURY, was absent in ABCP aggregates and PLU aggregates but strongly expressed in ABCP+PLU aggregates (Fig. 2C). TBXT was only expressed in the TFAP2A-negative cells. We also examined a mesendoderm marker, EOMES, and endoderm markers, GATA6 and SOX17 (*40*–*42*), and found that they were strongly induced only when ABCP and PLU cells were mixed together (Fig. 2D and fig. S1A). These results suggest that ABCP cells have the capacity to initiate mesendoderm specification of PLU cells. Next, to confirm the origins of the cells in ABCP+PLU aggregates, we made aggregates with PLU cells expressing green fluorescent protein (GFP). We observed the clear segregation of cells according to GFP expression (Fig. 2E). The amnioblast marker, ISL1, was evident in GFP-negative cells (Fig. 2, E and F, and fig. S1B), suggesting that the amnioblast-like state was maintained in ABCP cells. Moreover, we also detected TBXT exclusively in the GFP-positive cells of ABCP+PLU aggregates (Fig. 2, E and F, and fig. S1B), showing that only PLU cells are responsive to the gastrulation-inducing signal from ABCP cells. Together, our results suggest that ABCP-treated cells stimulate pluripotent epiblast cells to differentiate into mesendoderm lineages thereby demonstrating their functionality as AE.

### Single-cell RNA sequencing analysis on ABCP culture reveals the developmental trajectory of amniotic ectoderm

**Fig. 3.**
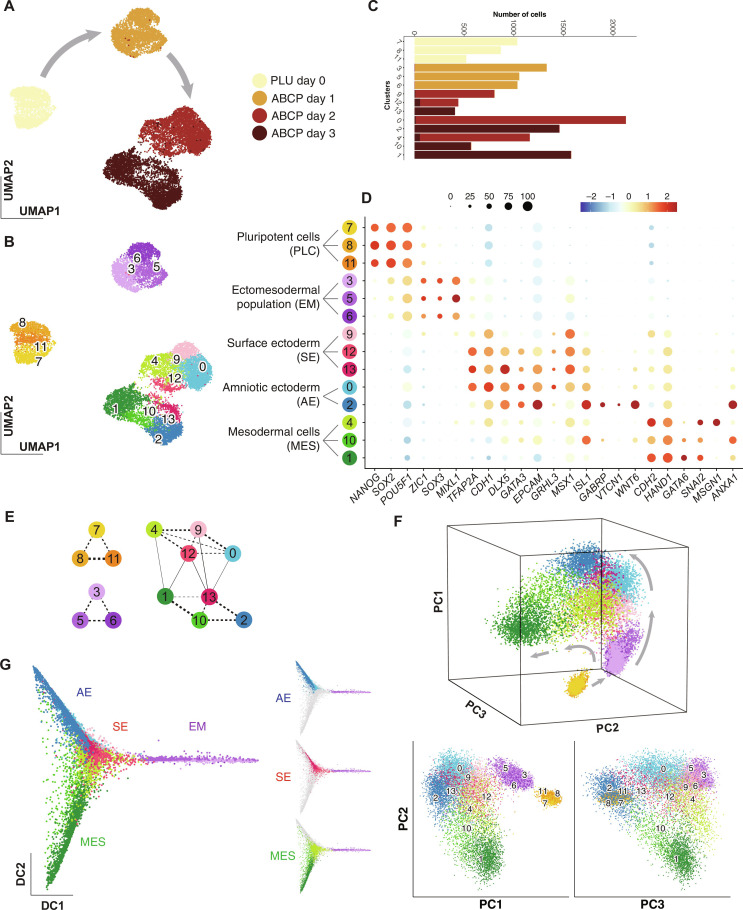
Dimensionality reduction projections and clustering of scRNA-seq analysis on ABCP low-density culture. (**A** and **B**) UMAP summarizing all 14,411 cells from pluripotency (day 0) and ABCP (day 1, 2, and 3) cultures. (A) Colors indicate collection days and gray arrows indicate chronological transitions. (B) Colors indicate cluster numbers. Hereafter, the same color scheme is used to indicate these clusters. (**C**) Cell counts per cluster. Colors indicate the collection days as in (A). (**D**) Bubble plot showing the variation in expression (ratio of cells and amplitude) of marker genes well characterized across the annotation groups. (**E**) Partition-based graph abstraction analysis on all the cells of the 14 clusters. Lines between clusters indicate pairwise similarities. The thickness of the lines reflects the connectivity values from the analysis. Values less than 0.1 are not shown. Dotted lines represent the similarities between clusters from the same collection days. (**F**) principal components analysis (PCA) plot of the 14,411 cells indicating the two directions of developmental trajectories (gray arrows) over the period of ABCP culture. Cluster numbers are indicated on the two-dimensional plots. (**G**) Diffusion map of ectomesodermal (EM), SE, AE, and mesodermal cells (MES) clusters.

To obtain insights into the in vitro AE differentiation at single-cell resolution, we performed scRNA-seq analysis on hPSCs grown in ABCP condition for 0, 1, 2, and 3 days as shown in (Fig. 1E). A total of 14,411 cells passed through quality controls and were segregated by days of collection using Uniform Manifold Approximation and Projection (UMAP) (Fig. 3A). Across this UMAP, we defined 14 clusters using a resolution of 1.0 based on the Louvain algorithm (Fig. 3B). These clusters were annotated into five groups—pluripotent cells (PLCs), ectomesodermal population (EM), SE, AE, and mesodermal cells (MES) (Fig. 3B). Clusters 7, 8, and 11 were from the pluripotency maintenance medium and annotated as PLC (Fig. 3, A to C). These clusters consisted of cells expressing *NANOG*, *SOX2*, and *POU5F1* with low expression of differentiation markers (Fig. 3, B and D, and fig. S2). Clusters 3, 5, and 6 correspond to differentiation day 1 and were annotated as EM (Fig. 3, A to C). Cells in these clusters were enriched for ectodermal and mesodermal genes, such as *ZIC1*, *SOX3*, and *MIXL1* (*43*–*45*), and started to express NNE markers, such as *TFAP2A* and *CDH1* (Fig. 3, B and D, and fig. S2) (*34*). Clusters 9, 12, and 13 were characterized by activated general NNE markers, especially *TFAP2A*, and low expression levels of AE markers (Fig. 3, B and D, and fig. S2). Furthermore, they were marked by the cells expressing SE marker genes, *GRHL3* and *MSX1* (*30*, *34*, *37*, *44*–*46*), and therefore were annotated as SE. Two large clusters featured by enhanced expression of AE genes (0 and 2) were annotated as AE (Fig. 3, A to D). *ISL1* was highly and broadly expressed within these clusters, while *GABRP* and *VTCN1* were more specific for subpopulations in clusters 0 and 2 (fig. S2). *WNT6* expression was observed in a part of cluster 0 and in the entire cluster 2. Last, clusters 4, 10, and 1 were highlighted by mesoderm markers, such as *CDH2*, *HAND1*, *GATA6*, and *SNAI2*, and thus annotated as MES (Fig. 3, B and D, and fig. S2). According to the previous scRNA-seq analyses of a human gastrula (*32*), cells in cluster 4 (ABCP day 2) expressed *MSGN1* and thus resembled the emerging mesoderm, whereas cells in clusters 1 and 10 (ABCP day 3) were enriched for *ANXA1*, suggesting their similarity to extraembryonic mesoderm. MES clusters were dissimilar from the other day-2 and day-3 clusters for the lack of general NNE gene expression, such as *TFAP2A*, *CDH1*, and *EPCAM*. To evaluate similarities between clusters, we performed partition-based graph abstraction (PAGA) analysis (Fig. 3E) (*46*). Clusters consisting of the cells collected on the same day (Fig. 3C) resembled each other (Fig. 3E, dotted lines). Between day-2 and day-3 clusters, cluster 4 had the strongest connection with cluster 1, and clusters 9 and 12 were closest to cluster 13 (Fig. 3E, solid lines), validating the annotation grouping of these clusters based on the selected markers (Fig. 3D). Note that the cluster groups from ABCP days 2 and 3, i.e., SE, AE, and MES, do not consist of homogenous cell types but rather contain cells at the distinct stages of differentiation toward the annotated lineages.

We also visualized the covariation of expression across the cells in the principal component analysis (PCA) (Fig. 3F). The transition through days 0, 1, 2, and 3 was well captured by the variance explained on the PC1 × PC2 plane. In addition, the PC2 × PC3 dimension highlighted the trajectory from PLC through EM into two distinctive paths—SE/AE and MES directions (gray arrows). The PCA plot also displayed the SE clusters appearing between the two AE clusters, with more advanced cluster 2 at the end of the NNE trajectory. Cluster 1, rather than cluster 10, was placed at the end of the MES trajectory. Diffusion map analysis confirmed the separation between the SE/AE clusters and MES clusters (Fig. 3G). In the SE/AE branch, cluster 2 spread distally from EM clusters, but clusters 12 and 13 stayed in proximity to the EM clusters. These results suggest that ABCP culture induces NNE differentiation toward amnioblast, aside from mesodermal lineages, and that SE and AE populations resemble each other.

### Amniotic ectoderm differentiation relies on NODAL, BMP, and WNT signal pathways

We further examined the expression of markers for other known cell types in human embryos at the implantation and gastrulation stages (fig. S3A). Genes indicative of trophectoderm (*4*, *5*, *47*) were almost undetectable, such as *SLC28A3*, *ADAP2*, and *NR2F2*, or weakly expressed, such as *ENPEP*, *KRT7*, and *GATA2*, throughout differentiation. There was no clear up-regulation of trophoblast markers (*CGB3* and *SIGLEC6*) nor markers of syncytiotrophoblast or extravillous trophoblast (*CGA*, *SDC1*, *HLA-G*, and *LVRN*). Also, cells in ABCP culture did not develop the hypoblast or definitive endoderm profiles represented by *SOX17* and *FOXA2* (*48*–*50*). Moreover, mesendoderm markers, *EOMES* and *GSC* (*51*), showed very limited up-regulation in EM cells, and another definitive endoderm marker, *FOXA3* (*52*), was absent in ABCP culture. Together, these results confirm that cells differentiated in ABCP condition do not induce trophectoderm, hypoblast, nor definitive endoderm fates.

Next, we investigated the expression of ligands and downstream targets of the signaling pathways stimulated by the ABCP condition (fig. S3B). We found three different expression patterns of NODAL signal components (*53*, *54*). *LEFTY1* and *TDGF1* were enriched in PLC clusters, while *NODAL* and *CER1* were up-regulated in EM clusters from ABCP day 1. *TGFB1* and *LEFTY2* were up-regulated in MES clusters from days 2 and 3 of ABCP culture. These results may represent the dual functions of the NODAL pathway involved in the maintenance and differentiation of hPSCs. As suggested by its importance for both the formation and function of amnioblasts, BMP signaling (*55*, *56*) was highly active in the clusters from ABCP days 2 and 3. Both *BMP2* and *BMP4* started to express in ABCP day 1 onward, while *BMP2* was more abundant in early AE and early MES clusters, and *BMP4* was enriched in the late AE and entire MES clusters. Their expression levels were relatively low in the SE clusters. The downstream transcription factors, *ID1*, *ID2*, *ID3*, and *SKIL*, were strongly expressed in day 2, late SE, and late AE clusters, but relatively at low levels in the late MES clusters. *DUSP5* and *DUSP6* are the direct downstream targets of ERK signaling that encode phosphatases to settle down active ERK forming negative feedback (*57*). These transcripts were enriched in PLC clusters on day 0 and were shut down through the rest of the culture. This confirms that the MEK inhibition remains potent throughout the examined period of ABCP culture. Consistently, we also observed similar expression patterns of *SPRY1* and *SPRY2*, the FGF antagonists downstream of ERK activity (*58*). FGF ligands were, in general, not up-regulated except for *FGF4* and *FGF8*, which regulate the epithelial-to-mesenchymal transition and cell migration at gastrulation (*59*, *60*), in early SE and MES clusters. In line with the AE-specific activation of *WNT6* (Fig. 3D and fig. S2), *WNT4* was also specifically expressed in AE clusters, whereas *WNT5A* was rather enriched in MES clusters. The WNT-responsive genes, *AXIN2*, *DKK1*, *NKD1*, and *RNF43* (*61*), were up-regulated except for PLC clusters, indicating the activation of the canonical WNT pathway by CHIR in ABCP culture. Collectively, these results confirmed the signal responses triggered by supplements in ABCP condition.

### Cell lineages defined in ABCP culture are present in cynomolgus monkey embryos around neurulation

To validate the cell types defined in ABCP culture, we compared our in vitro scRNA-seq data with in vivo scRNA-seq data from primate embryos of comparable developmental stages. We used scmap, which identifies the closest cells (neighbors) in a reference scRNA-seq dataset for each individual cell from a query scRNA-seq dataset leveraging expression of highly variable genes (*62*). Because SE becomes obvious around neurulation, we used a recently published scRNA-seq dataset from cynomolgus monkey (*Macaca fascicularis*) embryos at Carnegie stages (CS) 8 to 11, when primitive streak development, neural tube patterning, and neural crest differentiation take place (*63*) as the reference. This dataset contains anterior epiblast/ectoderm (m.ECT), early and late SEs (m.eSE and m.lSE, respectively), and extraembryonic mesoderm (m.exeMES) (fig. S4A). Although one cluster was originally annotated as amnion (m.AM), the primate AE markers are mostly expressed in m.lSE but not in m.AM (fig. S4B), and therefore, we interpreted the m.lSE cluster as AE rather than the m.AM cluster. Supporting this interpretation, nonneural ectodermal cells containing the AE population in a human gastrula at CS7 (*32*) were projected onto m.eSE and m.lSE but not m.AM (fig. S4C). Next, we mapped our scRNA-seq clusters onto the UMAP of the monkey dataset (fig. S4D). scmap projection revealed that differentiation in ABCP culture traced paths from m.ECT toward ectoderm and mesoderm lineages. Cells in PLC were projected mostly in m.ECT population, which corresponds with epiblast cells not going through mesendoderm specification. Neighbors of EM were found in caudal mesoderm (m.cauMES) and posterior epiblast (m.EPI) as well as m.eSE and m.ECT. About half of both SE and AE populations were akin to m.eSE, while the ratio of query cells projected to m.lSE increased from 8 to 44% between SE and AE populations. In contrast to these ectodermal lineages, the majority of cells in MES were projected onto mesodermal populations including yolk sac mesoderm (m.ysMES), lateral plate mesoderm (m.lpMES), and m.exeMES. Overall, these results validated the annotation of the populations in ABCP culture in an in vivo context.

### Extraembryonic mesoderm rises from a primed pluripotent state

**Fig. 4.**
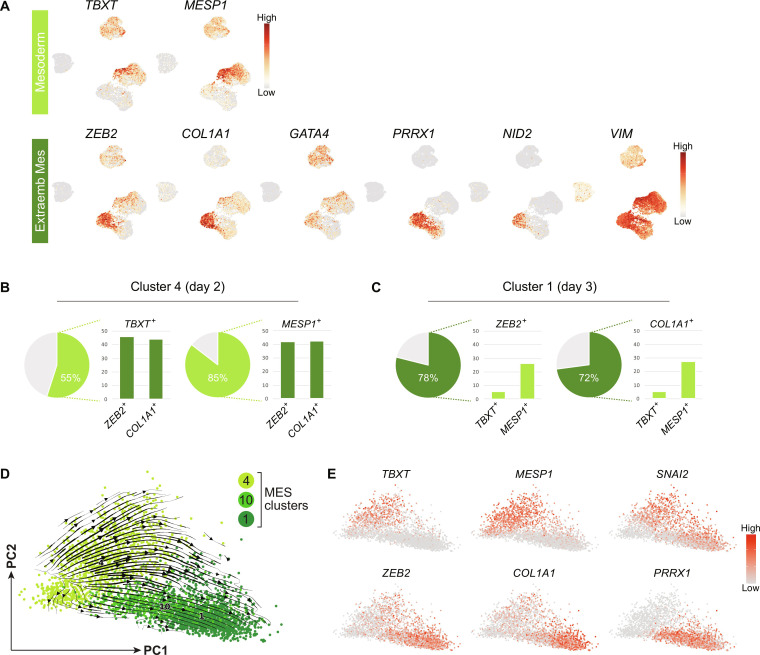
Embryonic and extraembryonic mesoderm genes in the MES trajectory. (**A**) Embryonic and extraembryonic mesoderm marker expression on the UMAP with the 14 clusters. See Fig. 3B for cluster locations. (**B** and **C**) Coexpression analysis of *TBXT*, *MESP1*, *ZEB2*, and *COL1A1* in clusters 4 and 1. (**D**) RNA velocity summary overlapped on a PCA plot of clusters 4, 10, and 1. Arrows indicate trajectories inferred using scVelo. (**E**) Marker gene expression overlapped on the PCA plot of clusters 4, 10, and 1.

Although the majority of cells cultured in ABCP condition for 2 days or more presented NNE profiles (Fig. 3, B and D), the unexpected appearance of MES (cluster 4 from day 2; clusters 10 and 1 from day 3) drew our attention. A recent study demonstrates that human preimplantation epiblast gives rise to extraembryonic mesoderm without expressing embryonic mesoderm genes, unlike mouse embryos where extraembryonic mesoderm emerges after gastrulation (*6*). Hence, we analyzed embryonic and extraembryonic mesoderm gene expression in our scRNA-seq data (Fig. 4A). Embryonic mesoderm genes, *TBXT* and *MESP1*, were broadly expressed among EM clusters on day 1 and became enriched in cluster 4 on day 2 (Fig. 4A, top). Similarly, *MIXL1* exhibited a clear increase on day 1 and remained expressed in cluster 4 (fig. S2). These genes substantially decreased on day 3 of ABCP culture. We also explored known markers of human extraembryonic mesoderm (*6*, *28*, *64*–*66*). In line with the suggested extraembryonic mesoderm identities of clusters 1 and 10 (Fig. 3D and fig. S2), *ZEB2*, *COL1A1*, *GATA4*, *PRRX1*, and *NID2* were highly up-regulated in these clusters but to limited degrees in cluster 4 (Fig. 4A, bottom). *VIM* expression was widely observed among day-2 and -3 cells as reported in both extraembryonic mesoderm and amnioblasts. Among these markers, *ZEB2* and *COL1A1* were also expressed in cluster 4. Thus, we examined their coexpression with embryonic mesoderm markers, *TBXT* and *MESP1*, in cluster 4 and found that about 45% of the *TBXT*- and *MESP1*-positive populations also expressed *ZEB2* or *COL1A1* (Fig. 4B). We also inspected the coexpression of these genes in cluster 1 and found that *MESP1* was expressed about 25% of *ZEB2*- and *COL1A1*-positive populations, while *TBXT* expression was low in these populations (Fig. 4C). These results suggest that clusters 4 and 1 contain cells expressing both embryonic and extraembryonic mesoderm markers.

To infer the differentiation trajectory of the extraembryonic mesoderm, we performed RNA velocity analysis on clusters 4, 10, and 1 using scVelo (*67*). On the PCA plot with the selected clusters, the pseudo-time arrows indicated two major trajectories from clusters 4 to 1 (Fig. 4D). The first path traverses the spreading cluster 4, where the cells expressing *TBXT* and *MESP1* (Fig. 4E) into cluster 1. The second path traverses a crowd of cluster 10 cells presenting low levels of *TBXT* and *MESP1* (Fig. 4E). Together, our results suggest that primed hPSCs can differentiate to the extraembryonic mesoderm lineage and a proportion of these cells transiently express embryonic mesoderm genes.

### SE and AE lineages originate from sequential differentiation of NNE

**Fig. 5.**
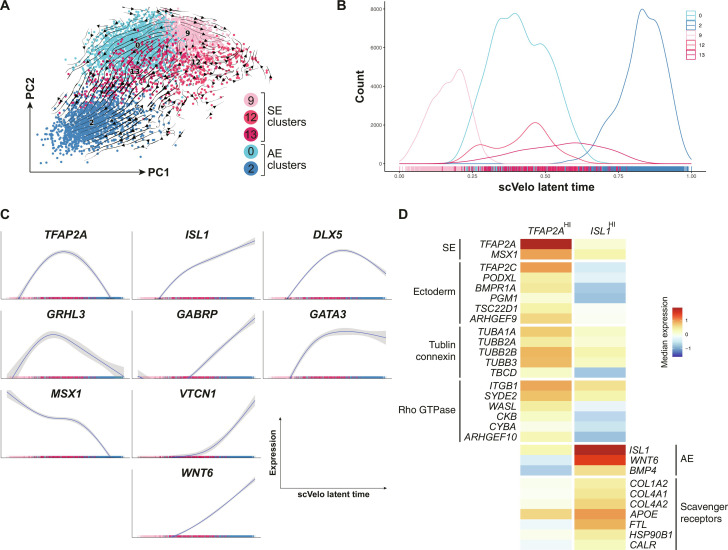
Gene expression dynamics during the SE/AE trajectory. (**A**) RNA velocity summary overlapped on the PCA plot of clusters 0, 2, 9, 12, and 13. Arrows indicate trajectories inferred using scVelo. (**B**) Density and rug plot showing the distribution of SE and AE clusters along latent time. (**C**) Dynamics of SE and AE marker genes along the latent time. (**D**) Heatmap of genes differentially expressed between *TFAP2A*-high and *ISL1*-high populations.

Since we identified both SE- and AE-like populations in ABCP culture, we further explored the processes behind their specification by performing trajectory analyses on the SE and AE clusters with scVelo. The RNA velocity analysis indicated a trajectory from cluster 9 (day-2 SE) to cluster 2 (day-3 AE) through clusters 0, 12, and 13 (day-2 AE, day-2 SE, and day-3 SE, respectively) (Fig. 5A). Clusters 12 and 13 also had a stagnation point (Fig. 5A). These results were consistent with the PCA plot and the diffusion map (Fig. 3, F and G). Along with the latent time axis obtained from the RNA velocity analysis (Fig. 5B), we depicted the dynamics of SE and AE marker genes and found distinctive expression patterns. The SE markers, *TFAP2A*, *GRHL3*, and *MSX1*, showed high expression levels at the early stage of the differentiation and rapidly decreased (Fig. 5C, left). In contrast, the AE markers, *ISL1*, *GABRP*, *VTCN1*, and *WNT6*, kept increasing toward the end of the trajectory (Fig. 5C, middle). *DLX5* and *GATA3* showed an intermediate pattern (Fig. 5C, right). These results were largely reproduced by an alternative trajectory search pipeline based on Monocle 3 (fig. S5, A to C) (*68*).

These expression dynamics indicated that the expression of SE markers peaks in the middle of the trajectory and declines when AE genes become active (Fig. 5C). To highlight the differences between these cellular states, we extracted subsets of cells highly expressing *TFAP2A* or *ISL1* along the trajectory. Consistently, marker gene analysis picked up SE and AE profile genes for *TFAP2A*-high and *ISL1*-high subpopulations, respectively (Fig. 5D). In addition, gene enrichment analysis detected specific enhancement of the ectodermal differentiation pathway, including *TFAP2C*, the pioneer transcription factor in early SE specification (*69*), in *TFAP2A*-high population (Fig. 5D, left). This subpopulation was also marked by tubulin folding and connexin transportation components and Rho guanosine triphosphatase (GTPase) signaling. In contrast, the *ISL1*-high population was enriched for collagen variants and scavenger receptors (Fig. 5D, right). These analyses suggest that the NNE commitment progresses through a state with enhanced expression of SE genes before activation of the AE gene program, which could confer competence for epidermal differentiation in the embryo proper.

To investigate the NNE trajectory in vivo, we applied scVelo to the related clusters in the cynomolgus monkey dataset (m.EPI, m.ECT, m.eSE, m.lSE, and m.AM) (*63*). The coherent velocity fields indicated the dynamics from m.ECT cluster through m.eSE cluster into m.lSE cluster (fig. S5D), which is in line with the trajectory described above in vitro (Fig. 5A). In contrast, transitions through m.AM cluster were not supported by solid velocity streamlines, confirming that m.AM is not on the NNE trajectory. Moreover, the marker gene dynamics along the estimated pseudotime revealed the transient and continuous up-regulations of SE markers and AE markers, respectively (figs. S4B and S5, E and F), as observed in our in vitro human dataset (Fig. 5C and fig. S5C). Together, these results confirm the sequential differentiation of NNE through SE to AE during early monkey development.

### Cell density directs cell fate decisions between amniotic and surface ectoderms

**Fig. 6.**
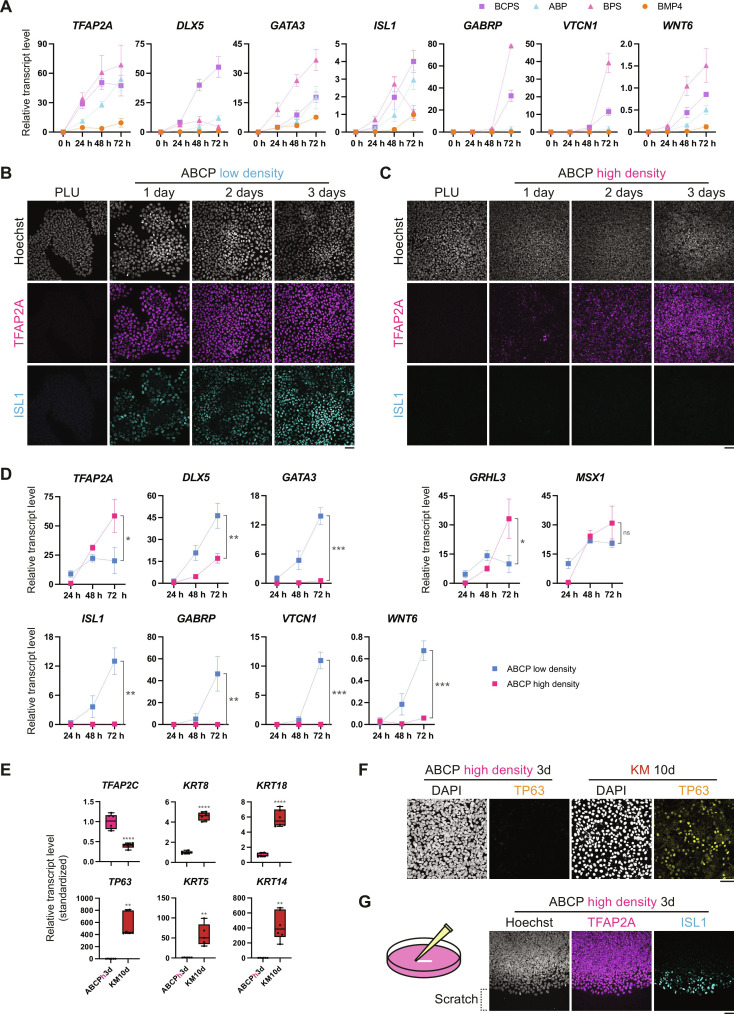
Investigation of ABCP culture derivatives on surface ectoderm induction. (**A**) Dynamic quantitative gene expression analyses over 3 days of ABCP culture variants. Dots and error bars represent means and SDs, respectively. *n* = 3. BCPS: BMP4, CHIR, PD, and SB; ABP: Activin A, BMP4, and PD; BPS: BMP4, PD, and SB. (**B** and **C**) Immunocytochemistry of ABCP cultures at original, low-density, and high-density conditions. Scale bars, 50 μm. (**D**) Dynamic quantitative gene expression analyses over 3 days of ABCP culture with low and high seeding densities. Dots and error bars are as in (A). To compare the expression levels of ABCP low- and high-density conditions at the 72-hour time point, the Student’s *t* test was performed. ns: *P* > 0.05, *P < 0.05, **P < 0.01, and ***P < 0.001. *n* = 3. (**E**) Quantitative gene expression analyses of cultures in ABCP high-density and following keratinocyte conditions. Box-plot elements: The center line is the median; box limits are upper and lower quartiles; whiskers are minimum and maximum. Student’s *t* test and Mann-Whitney test were performed on the basis of the results of the Kolmogorov-Smirnov test. **P < 0.01, ****P < 0.0001. *n* = 5 or 6. KM, keratinocyte culture medium. (**F**) Immunocytochemistry of ABCP high-density and keratinocyte cultures. Scale bar, 50 μm. (**G**) Immunocytochemistry of scratched ABCP high-density culture. Scale bar, 50 μm.

Quantitative RT-PCR and scRNA-seq analyses revealed that NNE and AE markers up-regulated concomitantly in ABCP culture (Figs. 1G and 3D), which is in agreement with the recent reports on the shared transcriptomic profiles of SE and AE (*24*, *32*). These observations prompted us to ask what distinguishes these lineages. In an attempt to define SE-inducing conditions, which solely up-regulate NNE markers without activating the amnioblast gene set, we replaced Activin A with SB and/or omitted CHIR in the ABCP condition (Fig. 6A). However, all the examined ABCP derivatives up-regulated the NNE markers together with the AE markers (Fig. 6A). The most efficient condition that up-regulated both NNE and AE markers consisted of BMP4, PD, and SB (BPS). In contrast to the previous report (*70*), the sole addition of BMP4 moderately up-regulated *TFAP2A*, *GATA3*, *ISL1*, and *WNT6* in our system. Together, these results indicate that neither NODAL nor WNT signaling is critical to selectively up-regulate NNE genes.

In coimmunostaining analysis, we noticed that TFAP2A precedes ISL1 expression in the areas densely packed with cells at the centers of colonies (Fig. 6B). Thus, we hypothesized that high confluence induces hPSCs to differentiate toward SE. To test this hypothesis, we differentiated hPSCs in ABCP condition with a high seeding density (200,000 cells/cm^2^: ~10 times higher than the previous conditions) that completely covered the culture surface with cells at the onset of differentiation (Fig. 6C). In this condition, most of the cells expressed TFAP2A protein in 3 days of differentiation, whereas ISL1 protein was undetectable throughout the differentiation (Fig. 6C). These results were reproduced by quantitative RT-PCR analyses (Fig. 6D). Notably, *TFAP2A* expression was boosted in the ABCP high-confluency condition with limited up-regulation of *DLX5* and *GATA3*. Moreover, the SE markers, *GRHL3* and *MSX1*, were up-regulated in the ABCP high-density condition. Of note, all the examined amnioblast markers, *ISL1*, *GABRP*, *VTCN1*, and *WNT6* were strongly suppressed. These results suggest that cell density plays a crucial role in the cell fate decision between SE and AE.

To further demonstrate their SE nature, we investigated the potential of the cells cultured in ABCP high-density condition to differentiate into keratinocytes, which represent a key cell type in the skin. Cells differentiated in ABCP high-density condition for 3 days were grown in keratinocyte culture medium for an additional 10 days (Fig. 6E). While SE marker *TFAP2C* decreased, simple epithelial markers *KRT8* and *KRT18* (*30*) increased over the course of the differentiation. Moreover, TP63, the master regulator of keratinocyte differentiation (*69*), was up-regulated (Fig. 6, E and F), together with epidermal markers, *KRT5* and *KRT14* (*69*). These data indicate that the cells grown in ABCP high-density conditions have the potential to further differentiate into cells in mature epidermises thereby demonstrating their functional capacity as epidermal progenitors.

To gain insights into mechanisms underlying the density effect, we performed wound scratch experiments. Confluent cellular sheets were scratched on the second day and incubated for another 24 hours in ABCP high-density culture (Fig. 6G). While the intact sheets were only positive for TFAP2A, the band of cells along the scratch loosened and expressed ISL1, demonstrating that exposure to a less dense environment is a key determinant in SE/AE fate choice and that SE cells are able to swiftly activate AE genes in the absence of neighbor cells. Gene enrichment analysis indicated differences between SE and AE in the Rho GTPase pathway (Fig. 5D), which is critical for cell motility and morphology (*71*). Thus, we inhibited Rho-associated protein kinases (ROCKs), major downstream effectors of Rho GTPases, during ABCP cultures. The presence of a ROCK inhibitor (Y27632: Y27) for 3 days of differentiation moderately down-regulated SE markers and up-regulated AE markers in both low- and high-density conditions (fig. S6, A and B). This implies a potential role of Rho GTPases in maintaining SE state.

## DISCUSSION

**Fig. 7.**
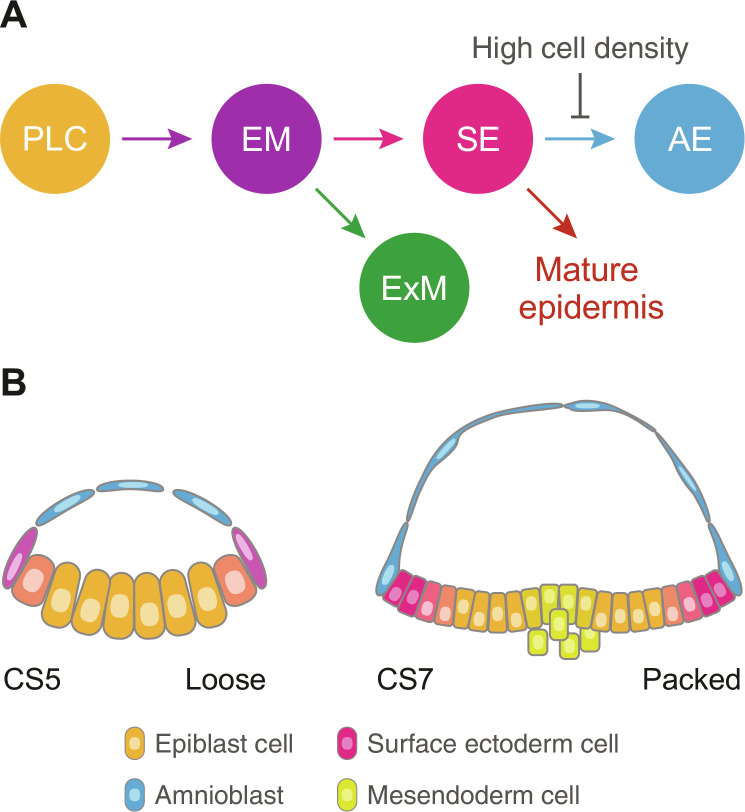
Sequential differentiation of nonneural ectoderm trajectory regulated by cellular density. (**A**) Schematic of the developmental trajectories in ABCP culture. ExM, extraembryonic mesoderm. (**B**) A model showing how human embryos could generate AE and SE by sequential differentiation and cellular density. Peripheral cells of the epiblast disc swiftly differentiate into amnioblasts as a loose sheet after implantation (left), while packed epiblast cells remain as SE around gastrulation (right). Intermediate colors indicate transitions between the cell states.

The biology behind the shared features between AE and SE in early human development remained elusive. Here, we developed a culture system to differentiate hPSCs into surface ectodermal and amnioblastic cells. We first demonstrated that ABCP culture at low seeding density generates amnioblast-like cells, which can provide gastrulation-inducing cues. scRNA-seq analysis identified populations displaying transcriptional profiles of SE and AE on a common developmental trajectory. We also examined combinations of signaling pathways to selectively up-regulate NNE genes, yet cell density was the only factor that influenced the cell fate choice between SE and AE. SE genes were specifically up-regulated without triggering AE genes when the differentiation was started at high seeding density. Together, these results indicate the sequential differentiation of SE and AE which is restrained by high cellular density (Fig. 7A).

Using published scRNA-seq data from cynomolgus monkey embryos, we confirmed that the cell lineages defined in ABCP culture resemble the related cell types found in vivo. Furthermore, we detected the SE-to-AE transition on the NNE trajectory in the monkey embryos as observed in our in vitro system, which implies that SE and AE emerge as a result of the sequential differentiation in vivo. The expansion of AE and initiation of gastrulation are observed around the same time in human and monkey embryos (*20*, *21*, *28*, *72*), suggesting that the specification of AE and SE occurs in close temporal proximity. Regarding cellular density, AE rises as a loose sheet with relatively small numbers of cells, while SE is formed as a continuous dense sheet of cells in human embryos (*73*, *74*), which is in agreement with the influence of cellular confluency we observed during in vitro differentiation. Moreover, transient gaps in the growing AE (*17*, *20*) might expose the epithelial progenitors on the edge to swiftly develop amnioblast features. All things considered, we propose a model in which AE and SE diverge from epiblasts according to cell density increasing as human embryos develop (Fig. 7B).

Although the mechanism by which cellular density affects SE/AE transition is currently unclear, the scratch experiment suggests that cell-cell junctions could play a regulatory role. Also, our gene enrichment search in scRNA-seq analysis and ABCP culture with the ROCK inhibitor indicated that the Rho GTPase signal contributes to SE/AE separation. Tight junctions between hPSCs regulate ligand-receptor interactions and thereby their susceptibility to differentiation cues (*75*). ROCKs are essential to assemble tight junctions of epithelial cells (*76*). Thus, it is suggested that cell density, cell-cell junctions, and Rho GTPases might work in concert to underscore the differentiation between SE and AE.

Our results also suggest an interesting involvement of mesodermal genes in amnioblast specification. *TBXT*, *MESP1*, and *MIXL1* transcripts were detected broadly on day 1 and partly on day 2 of ABCP culture. Transient up-regulation of TBXT during amnioblast specification was also described in vitro and in monkey embryos (*21*–*23*, *26*). Their rapid decrease on day 3 in ABCP culture might be explained by the inhibition of FGF/ERK signaling, which is necessary for embryonic mesodermal specification (*10*). Our scRNA-seq analysis also identified extraembryonic mesoderm-like cells expressing known markers, such as *ZEB2* and *COL1A1* on day 3 in ABCP culture, providing an example of extraembryonic mesoderm formation from primed hPSCs representative of the postimplantation epiblast. We detected an extraembryonic mesodermal development from the *TBXT*- and *MESP1*-positive population, which was not observed during the differentiation of naïve hPSCs into extraembryonic mesoderm (*6*). This supports the possibility that human extraembryonic mesoderm originates from the cells migrating before gastrulation (*7*, *28*) and/or from the cells ingressing at the beginning of gastrulation (*77*, *78*), both of which express mesoderm markers. Together, our study provides insights into the process of SE and AE specification, which will facilitate the development of methods to produce these tissues from hPSCs, while demonstrating the interest of our culture system as a model for the extraembryonic mesoderm formation in vitro.

## MATERIALS AND METHODS

### Maintenance and differentiation of hPSCs

Human embryonic stem cells (H9/WA09 line, WiCell) and induced pluripotent stem cells (FS13B line) were cultured on plates coated with vitronectin (10 μg/ml; Stem Cell Technologies). GFP-expressing FS13B cells were provided by the courtesy of C. M. Morell. For maintenance, cells were supplied daily with E6 media (*79*) supplemented with transforming growth factor–β (TGF-β; 2 ng/ml; Bio-Techne) and FGF2 (25 ng/ml; M. Hyvönen, Cambridge University) and were passaged every 5 to 7 days using 0.5 mM EDTA (Thermo Fisher Scientific) in phosphate-buffered saline (PBS; Thermo Fisher Scientific). Antibiotics were not used. For two-dimensional directed differentiation, hPSCs were dissociated using Accutase (Gibco) and plated as single cells in a pluripotency maintenance medium supplemented with 10 μM Y27632 (Selleck). The seeding density of low-density conditions was 5.0 × 10^4^ cells/cm^2^ for H9 and 2.1 × 10^4^ cells/cm^2^ for FS13B and that of high-density conditions was 2.0 × 10^5^ cells/cm^2^. Cells were counted by the Countess cell counter (Thermo Fisher Scientific). Two days after plating, the medium was changed to CDM-PVA medium (*80*) containing the supplements indicated for a given experiment. The medium was changed every 24 hours until cells were collected. ABCP condition contained Activin A (100 ng/ml; M. Hyvönen, Cambridge University), bone morphogenetic protein 4 [(BMP4) 10 ng/ml; Bio-Techne], 3 μM CHIR99021 (Tocris Bioscience), and 1 μM PD0325901 (Cambridge University). Where indicated, the following supplements were added: FGF2 (80 ng/ml), 10 μM LY294002 (Promega), 5 μM SCH772984 (Selleck), 10 μM SB431542 (Tocris Bioscience), and 0.1 μM LDN193189 (Sigma-Aldrich). See also Fig. 1 (A and E) for culture schedules. For keratinocyte differentiation, hPSCs were seeded on wells coated with Matrigel (1:100; Corning) at 1.6 × 10^5^ cells/cm^2^, such that the cells completely covered the culture surface at the onset of differentiation. Once the medium was changed to defined keratinocyte SFM (Thermo Fisher Scientific), half of the medium was replaced every day.

### Aggregation of ABCP and PLU cells

ABCP and PLU cells prepared on 12-well plates as described in Fig. 2A were washed once with PBS, treated with 400 μl of Accutase at 37°C for 5 min, and dissociated into single cells with 1000 μl of E6 medium containing 10 μM Y27632. Cell count was performed while the suspensions were spun down in 15-ml tubes at 300*g* for 3 min. Cell pellets were resuspended in E6 with Y27632 to reach a concentration of 1.0 × 10^4^ cells/ml, 40 μl of which was added per well of Repellent U-shaped 96-well plates (Greiner, 650970) to give a total of 400 cells per well. For ABCP+PLU aggregates, 200 cells from each of ABCP and PLU cell suspensions were dispensed per well. The plate was centrifuged at 300*g* for 3 min, and then incubated at 37°C and 5% CO_2_. Twenty-four hours later, 150 μl of E6 medium was added to each well.

### Quantitative RT-PCR

Total RNA was extracted from cells using GenElute Mammalian Total RNA Miniprep Kit (Sigma-Aldrich) and On-Column DNase I Digestion set (Sigma-Aldrich), or using RNeasy Mini kit and RNase-free DNase (Qiagen). Complementary DNA was synthesized from the RNA using random primers (Promega), dNTPs (Promega), RNAseOUT (Invitrogen), and SuperScript II (Invitrogen), or using RevertAid First Strand cDNA Synthesis Kit (Thermo Fisher Scientific). Real-time PCR was performed using KAPA SYBR FAST qPCR Master Mix (Kapa Biosystems) on QuantStudio 12 K Flex and QuantStudio 5 Real-Time PCR System machines (Thermo Fisher Scientific) or using SYBR Green PCR Master Mix (Thermo Fisher Scientific) on CFX Opus 384 Real-Time PCR System (Bio-Rad). Molecular grade water (Thermo Fisher Scientific) was used when necessary. Each gene expression level was normalized by the average expression level of *PBGD*. Primer sequences are shown in table S1.

### Immunostaining

For two-dimensional cultures, cells plated on vitronectin-coated round coverslips (Scientific Laboratory Supplies) were fixed with 4% paraformaldehyde (Alfa Aesar or Thermo Fisher Scientific) in PBS for 10 min at room temperature. After two PBS washes, cells were incubated with 0.25% Triton X-100 (Sigma-Aldrich) in PBS at 4°C for 15 to 20 min, followed by 0.5% bovine serum albumin (BSA; Sigma-Aldrich) in PBS at room temperature for 30 min. Cells were incubated with primary antibodies at 4°C overnight and secondary antibodies at room temperature for 1 hour. Each antibody incubation was followed by three washes with 0.5% BSA in PBS. Coverslips were preserved on glass slides (Corning) with ProLong Gold Antifade Mountant (Life Technologies) and CoverGrip Coverslip Sealant (Biotium).

For aggregates, staining was performed on Repellent U-shaped 96-well plates. To replace the solutions, the supernatant was carefully removed down to 50 μl once the aggregates were settled at the bottom of the wells and 150 μl of new solutions were added to each well. The aggregates were fixed with 4% paraformaldehyde in PBS at room temperature for 10 min, blocked with 0.5% BSA + 0.25% Triton X-100 in PBS at room temperature for 30 min, and incubated with primary antibodies at 4°C overnight or at room temperature for 1 hour, and with secondary antibodies at room temperature for 1 hour. Fixation and each antibody incubation were followed by three washes with PBS and 0.5% BSA in PBS, respectively. Then, the aggregates were transferred to 18-well chamber μ-slides (ibidi). BSA (0.5%)/PBS was removed and ScaleS4 solution (*81*) with 20% dimethyl sulfoxide was added.

Primary and secondary antibodies together with Hoechst33258 (10 μg/ml; Sigma-Aldrich) or DAPI (Cell Signaling Technology) were diluted in 0.5% BSA in PBS (see table S2). Images were taken using LSM 710 and LSM 980 inverted confocal systems (Zeiss) or Stellaris 8 (Leica). Ten optical sections were stacked for the section views in Fig. 2. To quantify GFP intensity, ISL1- and TBXT-positive cells were detected by the spot function in Imaris software in five and three ABCP+PLU_GFP aggregates, respectively.

### Single-cell RNA sequencing analysis

Human iPSCs (FS13B line) in pluripotency maintenance medium and ABCP low-density condition from the same sequence of differentiation were washed once with PBS and treated with Accutase for 5 min at 37°C for single-cell dissociation. RNA libraries were prepared using standard Illumina protocols for 10X Single-Cell GEX v3.

Raw fastq files were processed using Cell Ranger (v6.1.1); the alignment was performed against the GRCh38-3.0.0 version of the *Homo sapiens* reference genome; the quantification of mRNA expression and filtering of cells was conducted using default parameters. Further filtering applied on the raw expression matrix was based on upper and lower thresholds derived from the distributions of counts and features, and on the proportions of reads incident to mitochondrial DNA (mt%) and ribosomal genes (rp%), respectively. Cells with values outside these ranges (counts per cell/sequencing depth < 5000 or > 50000, number of features <2500 or > 7500, mt% > 15% rp% > 35%) were considered outliers and excluded from downstream analyses. After filtering, mitochondrial and ribosomal genes were excluded from the expression matrix, before normalization. The expression matrix was log-normalized using the NormalizeData function in the Seurat package (v4.2.0) (*82*). See fig. S7 for the summary quality control checks.

Dimensionality reductions [PCA followed by UMAP (*83*)], as well as clustering [Louvain algorithm (*84*)], were conducted in Seurat; the optimal number of clusters was selected on the basis of a stability analysis using the ClustAssess package (v0.3.0) (*85*). Following an assessment of the stability of clustering results, for subsequent steps, we focused on the 3000 most variable genes, across all cells in the dataset. The resolution of 1.0 induced stable and interpretable clusters. Marker genes were determined for each cluster versus the dataset complement, as well as all for pairwise cluster comparisons. These were identified on the basis of differential expression tests (in Seurat), i.e., genes with |log_2_(FC)| > 0.25, and adjusted *P* values, under a Benjamini-Hochberg multiple testing correction, less than 0.05. A gene set enrichment analysis was performed using the function gost from the package g:profiler2 (*86*). For each target cluster, all genes with expression >1 in at least 1 cell were used as a custom background set. The processed Seurat object was converted to a scanpy object, and PAGA was performed in scanpy with the function paga (*46*). A diffusion map was calculated for the subset of cells comprising clusters 0, 1, 2, 3, 5, 6, 9, 12, and 13 using the package destiny (v3.10.0) (*87*) with default parameters. Further gene-based cell subsetting was also performed; using a priori knowledge focused on two marker genes TFAP2A and ISL1, cells were sorted in descending order based on log_2_ expression of the marker genes and were split into three groups, corresponding to low, middle, and high expressions. Moreover, cells that did not express the marker genes (expression = 0) were filtered out to ensure the robustness of the downstream analysis.

RNA velocity analysis was performed in Python, using the scvelo package (v0.2.4) (*67*) with default parameters. The loom files were created using the velocyto package (v0.17.17), and the clustering, PCA, and UMAP calculated in Seurat were imported into Python to ensure consistency. The analysis was repeated on various subsets of cells (determined on resolution = 1.0); subset 1: clusters 0, 2, 9, 12, and 13 and subset 2: clusters 4, 10, and 1. The subset object was filtered and normalized with parameters: min_counts = 2, min_counts_u = 2, min_cells = 10, min_cells_u = 10, min_shared_counts = 2, min_shared_cells = 10 and subset_highly_variable = False; highly variable genes were retained. Moments for velocity estimation were computed with parameters: n_pcs = 30, use_rep = ‘X_pca’ and use_highly_variable = False. Transcriptional dynamics of splicing kinetics were learned in dynamical mode with default parameters. The package cellrank (v1.5.1) (*88*) was used to find terminal and initial states using cluster information from Seurat and parameter n_states = 1. Latent time was performed using the probabilities of the terminal and initial states.

The pseudo-time analysis was performed using the Monocle3 package (v1.3.1) (*89*) on the core set of clusters (0, 2, 9, 12, and 13). The starting point for the pseudo-time was determined using a voting scheme based on the following marker genes ISL1, GABRP, VTCN1, and WNT6. Cells expressing all four genes at the top 10 percentile were summarized as starting points. The pseudo-time was visualized on the core subset of clusters, on the PCA representation. For the nonlinear dimensionality reduction, we focused on the first three PCs. The learn_graph function was run with parameters: use_partition = FALSE and close_loop = FALSE. Cells were ordered starting from the selected root cells with default parameters. Since the selected genes were characteristic for an endpoint of the trajectory, the pseudo-time values of the cells were inverted; cells were ranked on the basis of the inverted pseudo-time values.

### Analyses with publicly available scRNA-seq dataset

Publicly available datasets were used for cross-comparisons. Raw counts and UMAP coordinates for the dataset of Tyser *et al.* (*32*) were downloaded from https://github.com/ScialdoneLab/human-gastrula-shiny. Processed raw data and raw fastq files for the dataset of Zhai *et al.* (*63*) were downloaded from Gene Expression Omnibus (GEO) (accession no: GSE193007). The function CreateSeuratObject(), from the Seurat package, was used to create a Seurat object with parameter min.cells = 3, i.e., genes expressed in less than three cells were excluded. Mitochondrial and ribosomal genes were filtered out from the new object. SCTransform() was used to normalize expression levels (*90*) with the following parameters: return.only.var.genes = FALSE, variable.features.n = 4000 (for Tyser dataset)/3000 (for Zhai dataset). RunPCA(), the exact implementation, was used to calculate principal components. RunUMAP() was used to calculate UMAP coordinates on the first 30 PCs. The UMAP coordinates were then replaced by the downloaded UMAP coordinates. For scmap, Seurat objects were converted to SingleCellExperiement objects (SingleCellExperiement package, version 1.20.1) (*91*). Common genes (i.e., genes found in the intersection) were used to subset the datasets and generate the input used for the scmap package [version 1.20.2; (*62*)]. The selectFeatures() function was used to identify the most informative/discriminative genes (parameter n_features =250). The indexCell() function was used with default parameters. The scmapCell() function was used to find neighbors and calculate cosine similarities, with default parameters.

For scVelo, reads R1, on raw fastq files, were trimmed using trim galore (version 0.6.10) (https://github.com/FelixKrueger/TrimGalore); 28 base pairs from the 5′ end were kept. Cell Ranger (v6.1.1) was used to align reads against the *M. fascicularis* genome (version 6.0) with default parameters. Loom files were created from the aligned BAM files using the velocyto package (v0.17.17). These were then combined using the scvelo package (v0.2.4) and subsetted to cells from the clusters defined in the original publication of Zhai *et al.* (*63*) as follows: “EPI,” “ECT’, “SE1,” “SE2,” and “AM.” PCs from the Seurat object and UMAP coordinates from the published paper were imported in Python; the subset object was filtered and normalized with the following parameters: min_counts = 2, min_counts_u = 2, min_cells = 10, min_cells_u = 10, min_shared_counts = 2, min_shared_cells = 10 and subset_highly_variable = False; highly variable genes were retained. Moments for velocity estimation were computed with parameters: n_pcs = 30, use_rep = ‘X_pca’ and use_highly_variable = False. Transcriptional dynamics of splicing kinetics were learned in dynamical mode with default parameters. The package cellrank (v1.5.1) (*85*) was used to find terminal and initial states using cluster information (from the Seurat partition) and parameter n_states = 1. Latent time was performed using the terminal and initial states.
