# Supplementary material for: Human surface ectoderm and amniotic ectoderm are sequentially specified according to cellular density
Source: Sci Adv. 2024 Mar 1;10(9):eadh7748. doi: 10.1126/sciadv.adh7748 (PMC10906920; doi:10.1126/sciadv.adh7748)
Supplement: Supplementary file 1 — Figs. S1 to S7 Tables S1 and S2 [file sciadv.adh7748_sm.pdf]

Science Advances
AAAS

Supplementary Materials for

Human surface ectoderm and amniotic ectoderm are sequentially specified according to cellular density

Shota Nakanoh *et al.*

Corresponding author: Shota Nakanoh, shota.nakanoh@babraham.ac.uk; Irina Mohorianu, iim22@cam.ac.uk; Teresa Rayon, teresa.rayon@babraham.ac.uk; Ludovic Vallier, ludovic.vallier@bih-charite

Sci. Adv. **10**, eadh7748 (2024)
DOI: 10.1126/sciadv.adh7748

This PDF file includes:

Figs. S1 to S7
Tables S1 and S2

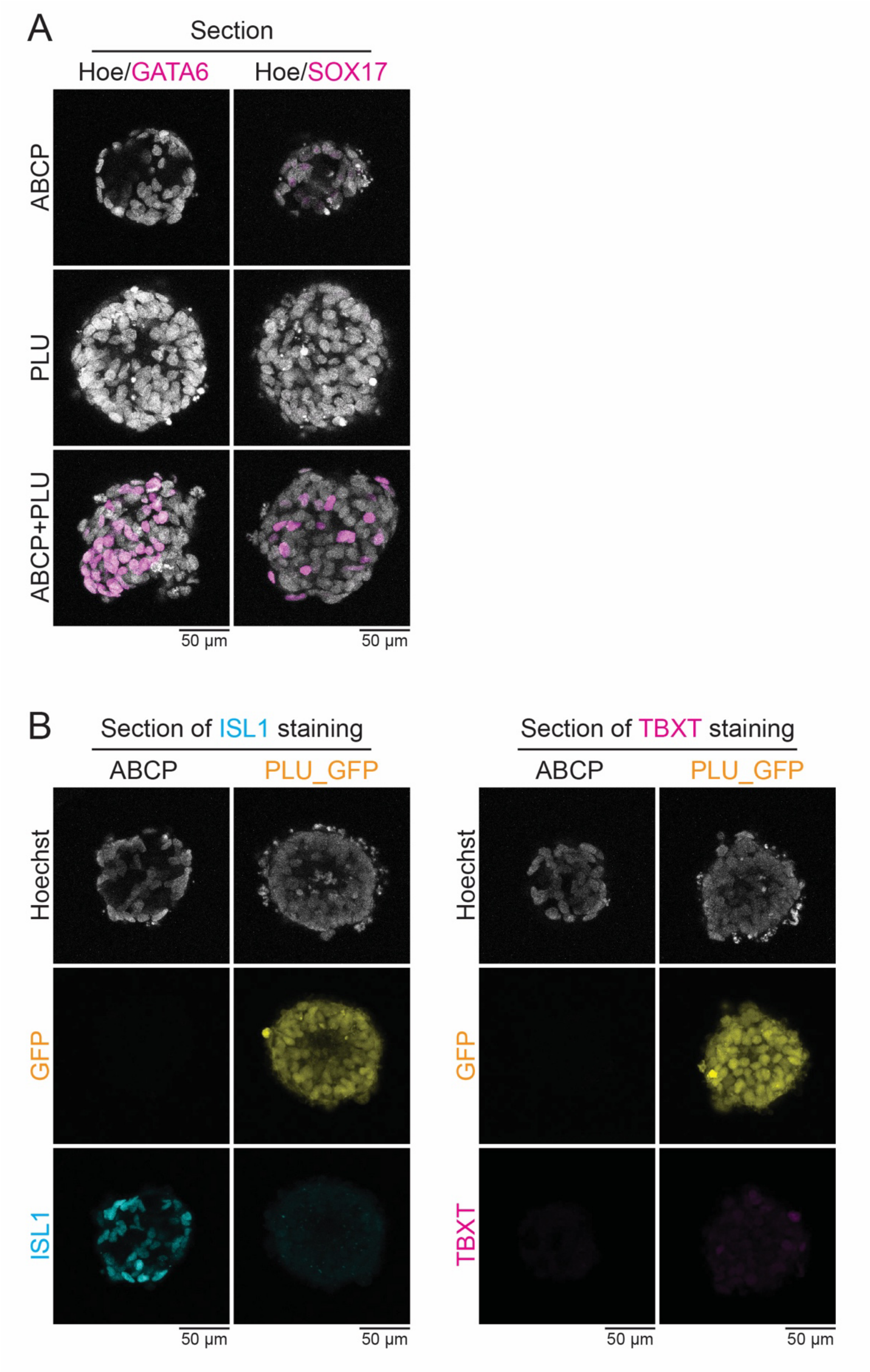

Fig. S1. Confocal microscopy on immunofluorescent staining of aggregates. (**A**) Stacked ten optical sections of ABCP, PLU and ABCP+PLU aggregates stained for GATA6 and SOX17. (**B**) Stacked ten optical sections of ABCP and PLU_GFP aggregates stained for ISL1 and TBXT.

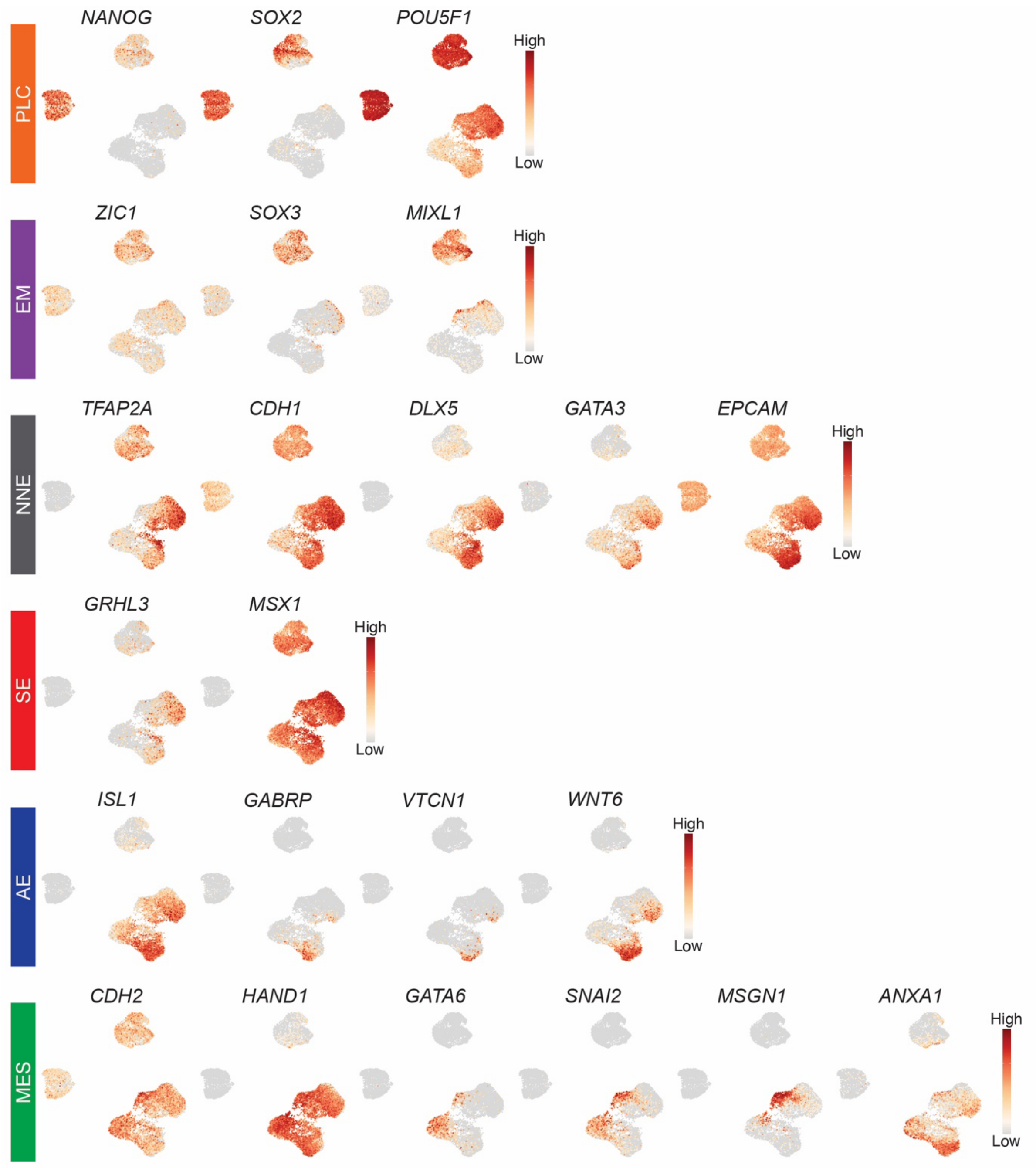

Fig. S2. Marker gene expression in ABCP scRNA-seq on UMAP. Marker genes characterizing the annotation groups.

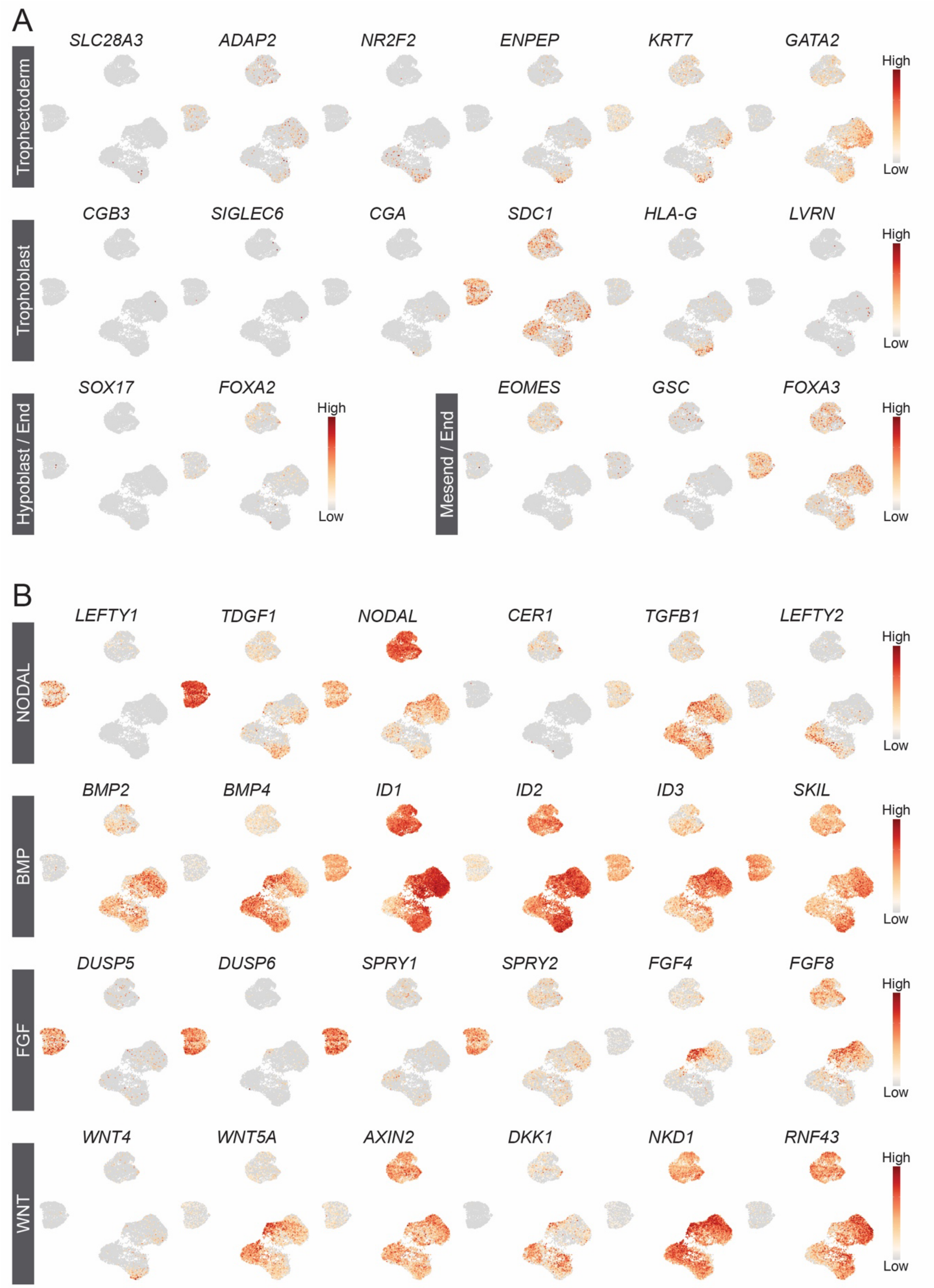

Fig. S3. Expression of markers for other cell types and signal pathway components in ABCP scRNA-seq. (**A**) Lineage specifying markers that did not upregulate in ABCP culture. (**B**) Ligands and downstream components of the four signaling pathways.

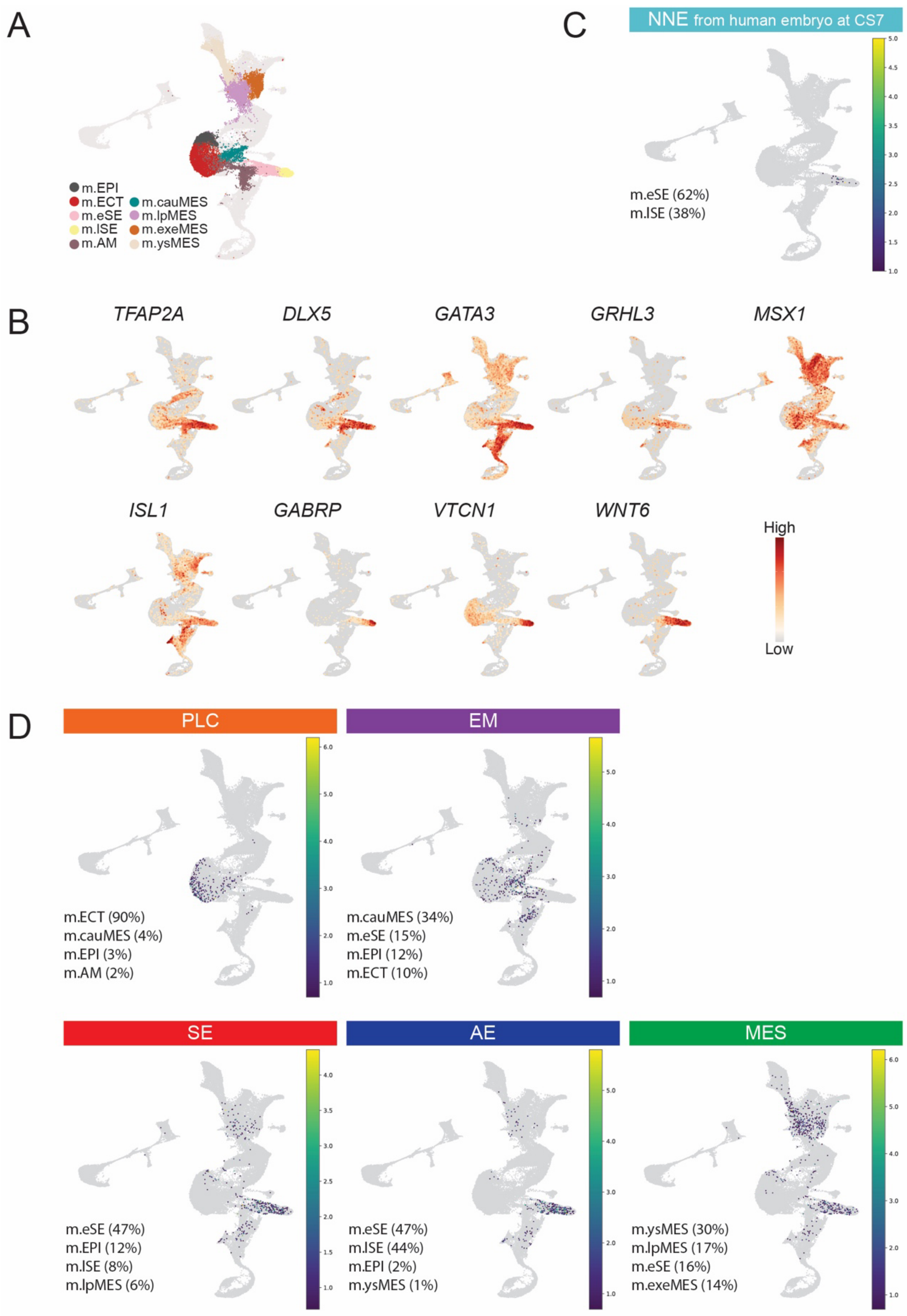

Fig. S4. Comparative transcriptomic analyses using a dataset from monkey embryos. (**A**) UMAP of published cynomolgus monkey neurula scRNA-seq highlighting relevant clusters. (**B**) SE and AE gene expression in the monkey data. (**C** and **D**) scmap projection of (C) NNE population from published human gastrula scRNA-seq and (D) cells from ABCP culture onto monkey scRNA-seq. Dots indicate neighbors. Dot color indicates number of cells projected onto the neighbor (natural log scale). Percentages are the ratios of query populations projected to the reference clusters. Up to the top four clusters with the greatest percentages are listed.

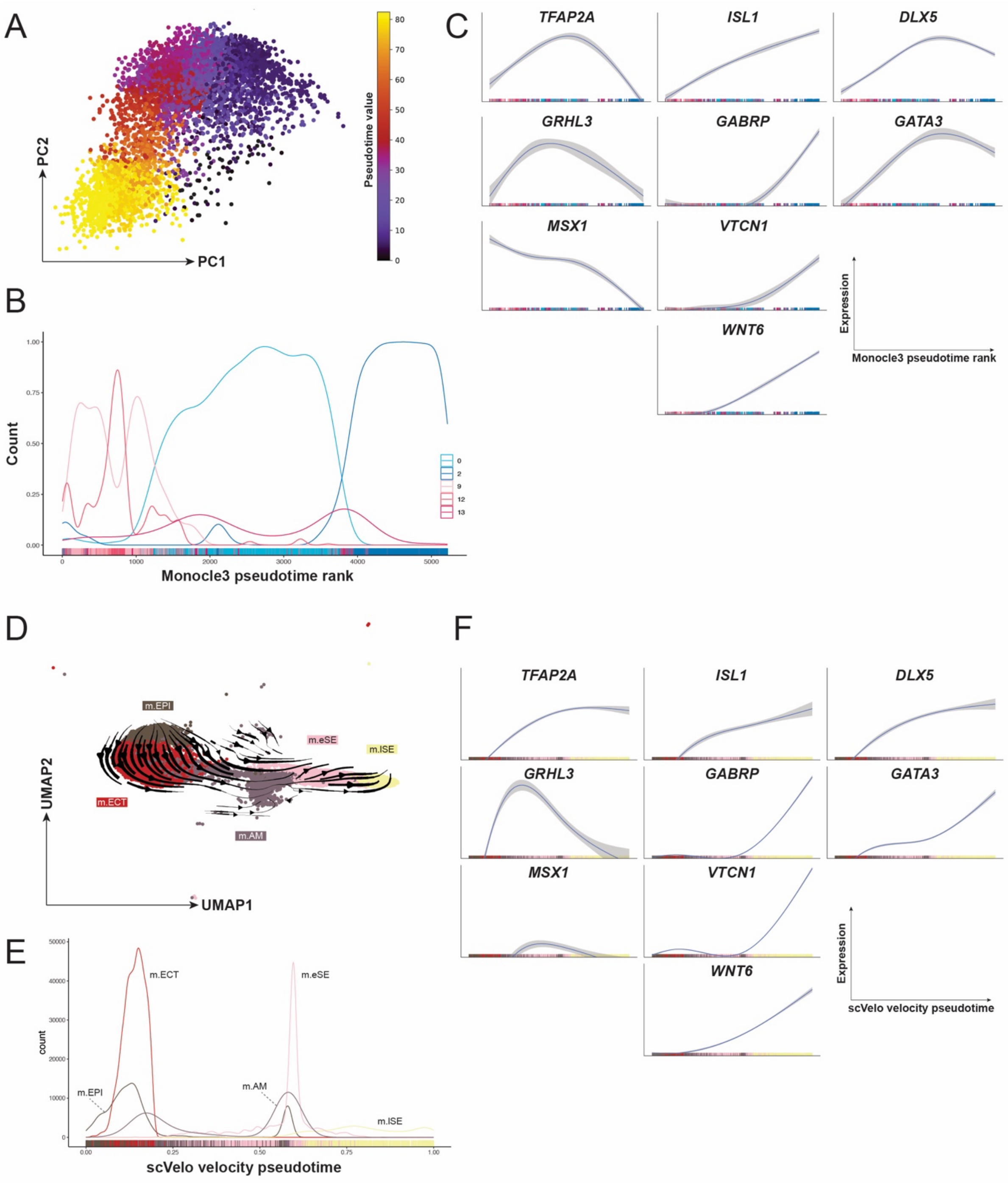

Fig. S5. Pseudotime search on the SE and AM trajectory. (**A**) Pseudotime color map on the PCA plot of clusters 0, 2, 9, 12 and 13 using Monocle3. (**B** and **C**) Density and rug plot, and marker gene dynamics along the Monocle3 pseudotime. (**D**) RNA velocity summary overlapped on the UMAP of selected clusters of the monkey *in vivo* data. Arrows indicate the trajectories inferred using scVelo. (**E** and **F**) Density and rug plot, and marker gene dynamics along the scVelo pseudotime estimated on the monkey clusters.

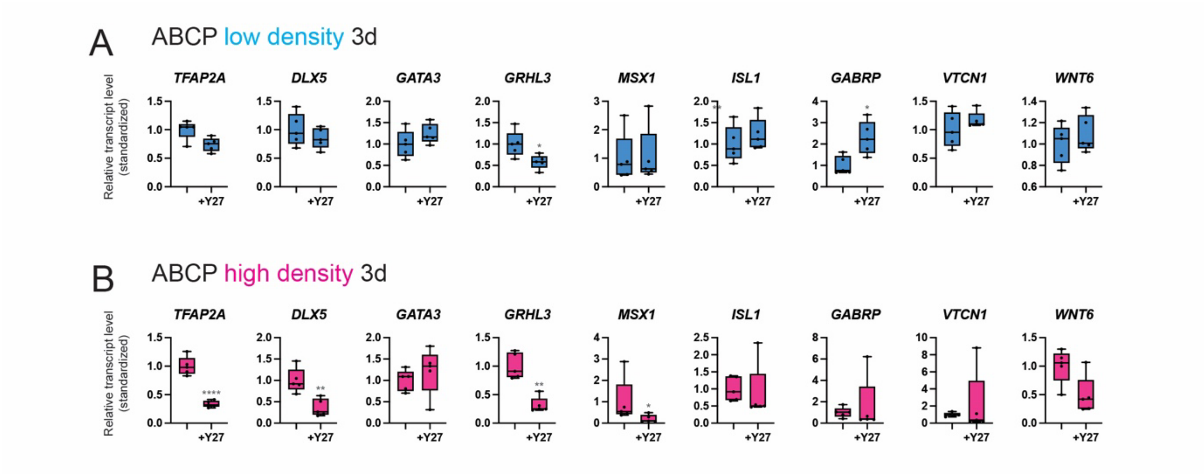

Fig. S6. Marker gene expression with a ROCK inhibitor. (**A** and **B**) Quantitative gene expression analyses of cells grown in ABCP culture for 3 days. Box-plot elements: center line is median; box limits are upper and lower quartiles; whiskers are minimum and maximum. Student's *t*-test and Mann-Whitney test were performed based on the results of Kolmogorov-Smirnov test. no mark: p-value > 0.05, *: p-value < 0.05, **: p-value < 0.01, ****: p-value < 0.0001. $n = 5$.

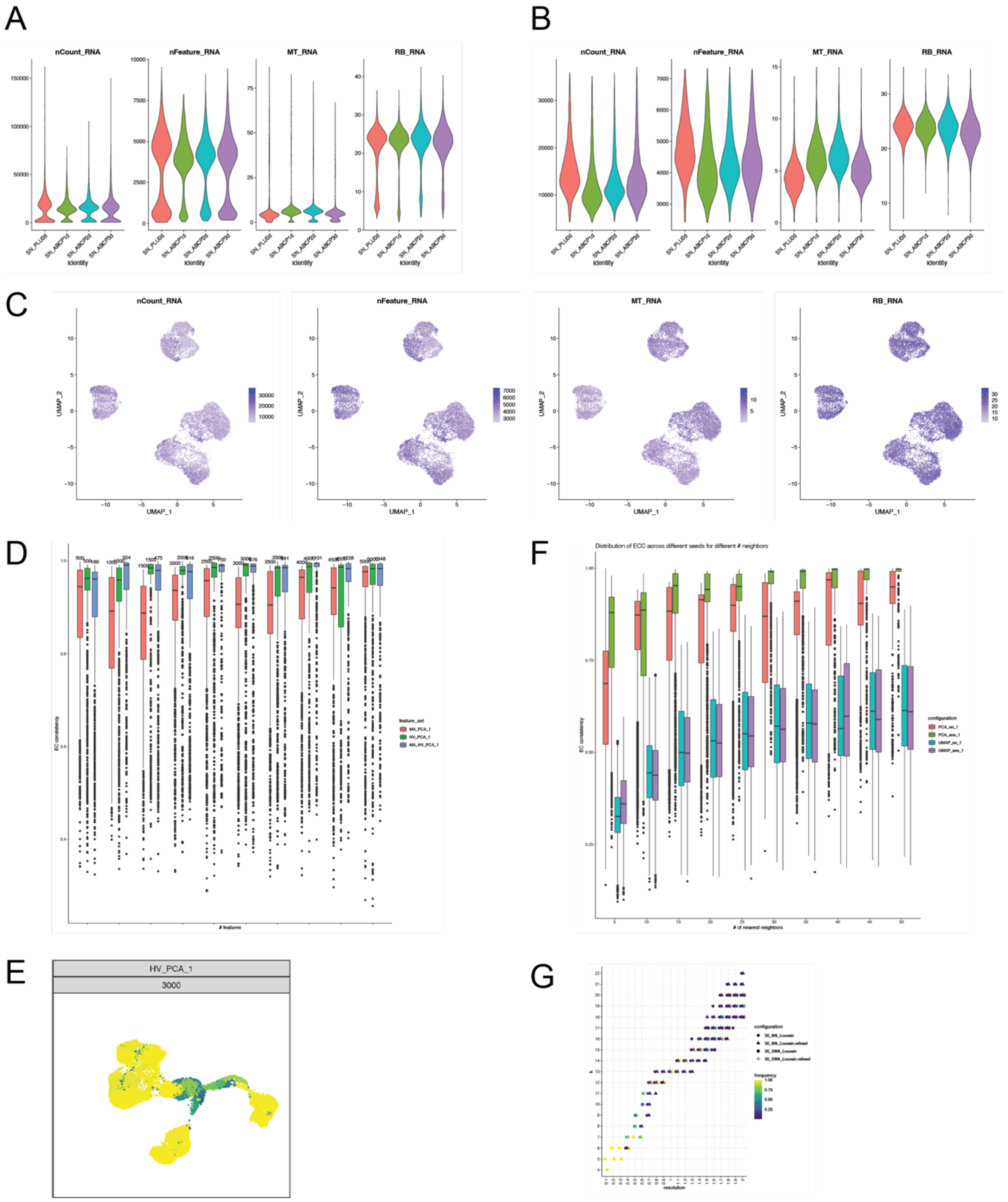

Fig. S7. Quality control of the scRNA-seq data. (**A**) Violin plots on the ncount, nfeature, %mt, %rp of the raw data. (**B**) Violin plots on the ncount, nfeature, %mt, %rp of the filtered data. (**C**) UMAPs for the ncount, nfeature, %mt and %rp. (**D**) Box plots of incremental variation in number and type of features assessed using Element-Centric Consistency Scores. (**E**) Distribution of Element-Centric Consistency scores represented on the UMAP created with top 3000 highly variable genes. (**F**) Box plots of incremental variation in number of neighbors assessed using Element-Centric Consistency Scores. (**G**) Scatter plot for assessing the number of clusters versus the resolution value evaluated using the Element-Centric Consistency score.

Table S1.
Primes for qPCR used in the study.

	Forward	Reverse
PBGD	GGAGCCATGTCTGGTAACGG	CCACGCGAATCACTCTCATCT
TBXT	TGCTTCCCTGAGACCCAGTT	GATCACTTCTTTCCTTTGCATCAAG
EOMES	ATCATTACGAAACAGGGCAGGC	CGGGGTTGGTATTTGTGTAAGG
TFAP2A	AGCTGCCAACGTTACCCTGCTCAC	GGCAGGAAATTCGGTTTCGCACACG
DLX5	AGCGCCACCAACCAGCCAGAGAAA	GTTTGTGTCAATCCCAGCGAGGCGG
GATA3	TCCTCCTCCTCTCTGCTCTTCGCT	AGGGGCGACGACTCTGCAATTCT
ISL1	AAGGACAAGAAGCGAAGCAT	TTCCTGTCATCCCCTGGATA
GABRP	TTTCTCAGGCCCAATTTTGGT	GCTGTCGGAGGTATATGGTGG
VTCN1	TCTGGGCATCCCAAGTTGAC	TCCGCCTTTTGATCTCCGATT
WNT6	GGCAGCCCCTTGGTTATGG	CTCAGCCTGGCACAACTCG
GRHL3	GGACCTCACTCCCCTTGAAAG	CAGTGGTGGGTAACAGGTAGC
MSX1	ACACAAGACGAACCGTAAGCC	CACATGGGCCGTGTAGAGTC
TFAP2C	CGCTCATGTGACTCTCCTGACATCC	TGGGCCGCCAATAGCATGTTCT
KRT8	GATCGCCACCTACAGGAAGCT	ACTCATGTTCTGCATCCCAGACT
KRT18	TCGCAAATACTGTGGACAATGC	GCAGTCGTGTGATATTGGTGT
TP63	AGAAACGAAGATCCCCAGATGA	CTGTTGCTGTTGCCTGTACGTT
KRT5	ATCTCTGAGATGAACCGGATGATC	CAGATTGGCGCACTGTTTCTT
KRT14	GACCATTGAGGACCTGAGGA	ATTGATGTCGGCTTCCACAC

Table S2.

Antibodies used in the study.

	Provider	Product #	Dilution
TFAP2A	Santa Cruz	sc-12726	1/100 or 1/200
ISL1 (Reconstituted at 0.2 mg/ml in PBS)	R&D	AF1837	1/100 or 1/200
NANOG (Reconstituted at 0.2 mg/ml in PBS)	R&D	AF1997	1/100 or 1/200
TBXT	abcam	ab209665	1/100 or 1/200
TBXT (Reconstituted at 0.2 mg/ml in PBS)	R&D	AF2085	1/100 or 1/200
EOMES (Reconstituted at 0.5 mg/ml in PBS)	R&D	MAB6166	1/100 or 1/200
GATA6	Cell Signaling Technology	5851	1/100 or 1/200
SOX17 (Reconstituted at 0.2 mg/ml in PBS)	R&D	AF1924	1/100 or 1/200
TP63	abcam	ab124762	1/100 or 1/200
Donkey anti-Mouse IgG 647	Invitrogen	A31571	1/1000
Donkey anti-Rabbit IgG 568	Invitrogen	A10042	1/1000
Donkey anti-Goat IgG 488	Invitrogen	A11055	1/1000
Donkey anti-Mouse IgG Plus 647	Invitrogen	A32787	1/1000
